# Bioactivity-Guided Fractionation of Dragon’s Blood Phenolic Extracts Reveals Loureirin D as a P2Y12 Inhibitor Mediating Antiplatelet Effects

**DOI:** 10.3390/ijms27010282

**Published:** 2025-12-26

**Authors:** Jiawen Peng, Peng Wang, Ying Chen, Xin Liao, Hui Guo, Pei Zhang, Jiange Zhang

**Affiliations:** 1 The Research Center of Chiral Drugs, Innovation Research Institute of Traditional Chinese Medicine, Shanghai University of Traditional Chinese Medicine, Shanghai 201203, China; 2 School of Pharmacy, Guizhou University of Traditional Chinese Medicine, Guiyang 550025, China; 3 State Key Laboratory of Discovery and Utilization of Functional Components in Traditional Chinese Medicine, Shanghai University of Traditional Chinese Medicine, Shanghai 201203, China

**Keywords:** thrombosis, ischemic stroke, platelets aggregation, P2Y12, ADP

## Abstract

Dragon’s Blood, from the Dracaena cochinchinensis plant, is known for enhancing blood circulation. Its main components are Dragon’s Blood phenolic extracts (DBE). To pinpoint the active DBE constituents that are effective against thrombosis and understand their mechanism of action, the PT-stroke model was employed to assess DBE’s antithrombotic effects on cerebral blood flow and platelet aggregation. This investigation demonstrates that DBE enhances cerebral blood flow and inhibits ADP-induced platelet aggregation in photothrombotic (PT) stroke models. An FeCl_3_-induced carotid artery thrombosis model was developed to test the antithrombotic activity of four DBE fractions. Through screening with this model, the ethyl acetate (EA) and methanol fractions were identified as the principal active components that effectively reduced thrombus weight and improved hemodynamics. Furthermore, the EA fraction was found to preserve the integrity of the blood–brain barrier. Phytochemical isolation allowed for the identification of compounds in the EA fractions, and UHPLC-MS was performed to characterize DBE and its active components in the bloodstream. In vitro ADP-induced platelet aggregation assays highlighted the active compounds. Through phytochemical analysis, Loureirin D (compound **17**) was identified as a predominant constituent present in plasma. In vitro assays revealed that compounds **1** and **17** possess strong antiplatelet activity, with Loureirin D being confirmed as a selective P2Y12 receptor antagonist via molecular docking and cellular thermal shift assays. These findings substantiate Loureirin D as a pivotal antithrombotic component in DBE and its potential as a P2Y12-targeting therapeutic agent for thrombosis treatment.

## 1. Introduction

Ischemic stroke—a cerebrovascular disorder marked by high rates of disability and mortality—represents a substantial threat to global public health [1]. The pathogenesis of this condition is primarily linked to thrombosis, which significantly contributes to disease progression via vascular occlusion [2].

Thrombus formation results in a reduction or complete cessation of cerebral blood flow, consequently depriving neural tissues of critical oxygen and nutrient supplies. Excessive platelet activation and aggregation constitute the primary mechanism underlying thrombus formation. Current therapeutic strategies predominantly involve the use of antiplatelet agents [3], including COX inhibitors (aspirin) and ADP receptor P2Y12 antagonists (clopidogrel and ticagrelor). However, the prolonged administration of these medications is associated with significant risks of hemorrhagic complications and thrombocytopenia [4]. This clinical challenge underscores the urgent necessity to develop novel platelet inhibitors that achieve a balance between therapeutic efficacy and improved safety profiles for ischemic stroke intervention [5].

Pharmacological validation studies have confirmed that traditional Chinese medicines (TCMs) with blood-activating and stasis-resolving pharmacodynamic properties exhibit significant antithrombotic therapeutic potential. Specifically, *Salvia miltiorrhiza* (Danshen) demonstrates cardiovascular efficacy by inhibiting platelet activation through the suppression of TXA2 production and ADP receptor antagonism [6]. Additionally, *Ligusticum chuanxiong* (Chuanxiong) exerts dual antithrombotic effects via the modulation of AA metabolism and downregulation of coagulation factor III expression [7].

Dragon’s Blood (Resina Draconis) is a red resin from *Dracaena cochinchinensis* (Lour.) S. C. Chen that has been used in traditional Chinese medicine. It promotes blood circulation, thus resolving stasis, reducing inflammation, alleviating pain, and facilitating tissue regeneration. Modern research has demonstrated its pharmacological effects in protecting the cardiovascular system [8,9] and nervous system [10], as well as promoting anti-tumor activity [11,12] and wound healing [13]. Its chemical constituents comprise phenols, terpenoids, and sterols, with phenolic compounds identified as the primary active components [14]. Longxue Tongluo Capsules, which contain Dragon’s Blood phenolic extracts (DBE) as the main ingredient, have been utilized for the treatment of ischemic stroke [15], atherosclerosis [9], and cardiovascular protection [16], demonstrating promising potential for clinical application. Although DBE have been utilized in traditional medicine for the treatment of thrombotic diseases, the precise pharmacological mechanisms underlying its antiplatelet activity remain poorly understood. A systematic identification of the active components in DBE would not only help to elucidate its scientific mode of action, but may also serve as a source of lead compounds for the development of novel therapies targeting arterial thrombosis.

This study focuses on the isolation and extraction of DBE compounds with varying polarities to identify the specific active constituents that inhibit platelet aggregation. The objective is to establish a robust theoretical and experimental foundation for the development of safer and more efficacious antithrombotic agents, potentially contributing to significant advancements in the treatment of arterial thrombosis.

## 2. Results

### 2.1. The Antithrombotic Effect of DBE

**Figure 1:**
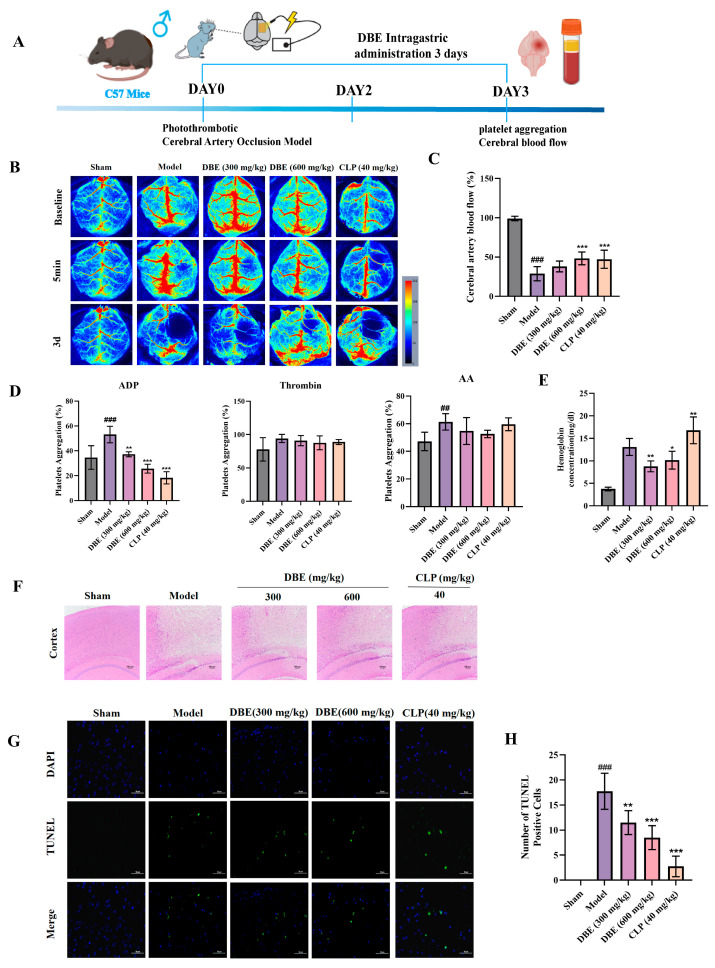
The antithrombotic and stroke-protective effects of DBE. (**A**) Schematic diagram of the experimental protocol for animals. (**B**) Representative images of CBF at Day 3 post-treatment in every group. (**C**) The statistical analysis of CBF for Figure 1B (*n* = 6). (**D**) Different agonists, such as ADP, thrombin, and AA, induce platelet aggregation (*n* = 6). (**E**) Intracerebral hemoglobin content (*n* = 6). (**F**) H&E staining was performed to observe pathological damage in mouse brain tissue. Scale bar = 100 μm. (**G**) TUNEL staining was performed on brain tissue sections of from different experimental groups. Scale bar = 50 μm. (**H**) Numerical analysis of Nissl staining with the ImageJ program (*n* = 4). Results are expressed as means ± SD. * *p* < 0.05, ** *p* < 0.01, *** *p* < 0.001 versus Model group, ## *p* < 0.01, ### *p* < 0.001 versus Sham group.

A PT-stroke model was developed to examine cerebral hemodynamic alterations in mice using laser speckle contrast imaging. Successful establishment of the model was verified by a 50% decrease in cerebral blood flow following photochemical-induced injury. Three days of oral administration of DBE significantly ameliorated cerebral ischemia (Figure 1B,C), with the treatment group exhibiting a 30% higher cerebral blood flow compared to the Model group, as determined by quantitative perfusion analysis. To elucidate the mechanism of action, we conducted in vitro platelet aggregation assays using multiple physiological agonists: adenosine diphosphate (ADP, 3 μM), arachidonic acid (AA, 1 mM), and thrombin (1 U/mL). DBE demonstrated selective inhibition of ADP-induced platelet aggregation while exerting minimal effects on AA- and thrombin-mediated pathways. This inhibitory effect was dose-dependent (Figure 1D). Considering the clinical concern regarding the hemorrhage risk associated with antiplatelet therapies, we quantified the cerebral hemoglobin content following treatment. Notably, DBE administration preserved physiological hemoglobin levels comparable to the Sham group, in contrast to the clopidogrel (CLP)-treated group (40 mg/kg), which exhibited significant HT (Figure 1E).

### 2.2. Neuroprotective Effects of DBE in Ischemic Stroke

Arterial thrombosis is the primary cause of ischemic stroke. To investigate the neuroprotective potential of DBE against thrombosis-induced cerebral infarction, we conducted a histopathological evaluation using HE staining and assessed apoptosis via a TUNEL assay in a PT-stroke model after three days of administration of DBE. HE staining revealed distinct histological profiles across the experimental groups. The Sham group exhibited preservation of intact cytoarchitecture with regularly arranged neurons and physiological cellular density. In contrast, the Model group displayed severe structural disorganization, characterized by cortical and striatal fragmentation, pronounced cellular edema, and nuclear pyknosis. Both DBE and clopidogrel significantly attenuated infarct volume and improved cellular morphology, as evidenced by decreased vacuolization and normalized perineuronal net spacing (Figure 1F). Of note, consistent results were obtained from the TUNEL analysis, wherein blue fluorescence labeled nuclei and green fluorescence indicated apoptotic cells (TUNEL-positive cells). Cells in the Sham-operated group exhibited minimal green fluorescence, whereas a high intensity of red fluorescence in the untreated Model group indicated a marked increase in apoptosis. Moreover, the intensity of green fluorescence was significantly lower in cells derived from animals treated with DBE (Figure 1G,H). These multimodal analyses provide robust evidence that DBE offer substantial neuroprotection against thrombosis-induced ischemic injury.

### 2.3. Analysis of the Antithrombotic Active Components in DBE

The phenolic extract of Dragon’s Blood exhibits remarkable structural diversity, primarily comprising flavonoids, phenylpropanoids, stilbenes, phenolic acids, and polyphenol polymers, which represent its principal bioactive components [14,17,18]. It has been confirmed that the phenolic extracts of Dragon’s Blood possess pharmacological effects, including anti-inflammation effects [19], anti-atherosclerotic properties [9], and anti-thrombosis activities [20,21,22]. Notably, these extracts have demonstrated remarkable efficacy in antithrombotic therapy. However, the specific antithrombotic components remain to be elucidated. This study aims to identify the specific antithrombotic components in DBE and to comprehensively elucidate their mechanisms of action in detail.

**Figure 2:**
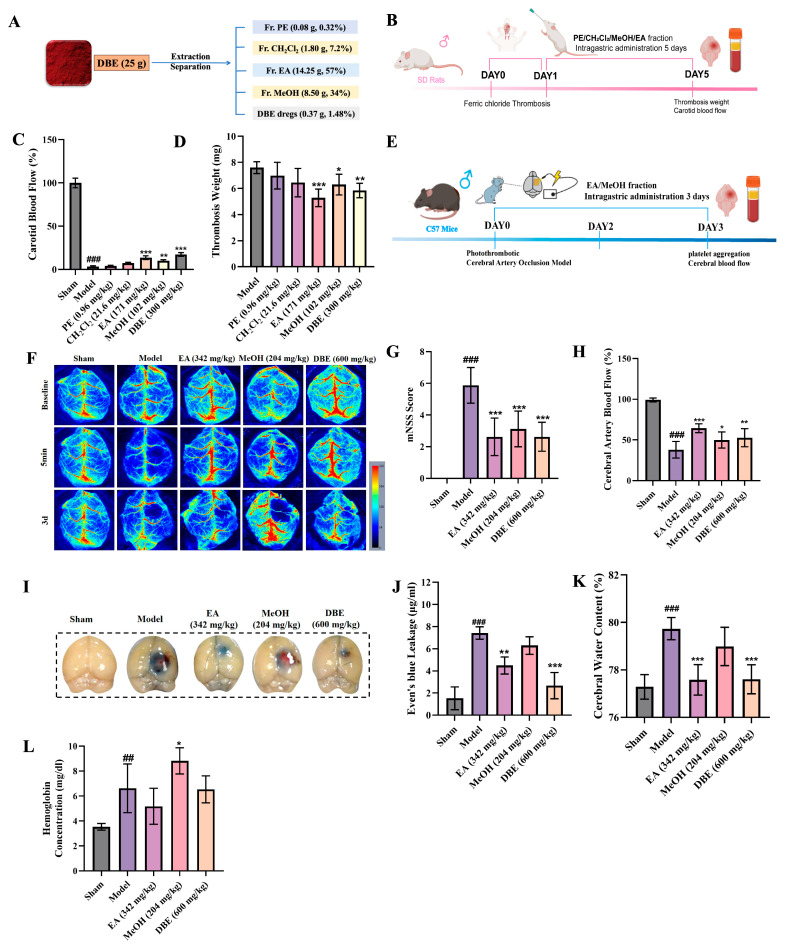
Evaluation of the antithrombotic activity and neuroprotective effects of DBE obtained using different polar solvents in the context of ischemic stroke. (**A**) Diagram illustrating the chromatography separation of DBE. (**B**) Schematic diagram of the thrombotic animal model in the experimental protocol. (**C**) Carotid artery blood flow measurement (*n* = 6). (**D**) Thrombosis weight (*n* = 6). (**E**) Schematic diagram of the experimental protocol for ischemic stroke animal models. (**F**) Assessment of neurological function scores in mice (*n* = 8). (**G**) Statistical analysis of CBF in Figure 2F (*n* = 10). (**H**) Representative images of CBF at 3 days post-injury in each group. (**I**) Representative Evans Blue staining images. (**J**) Quantitative analysis of Evans Blue extravasation in the ipsilateral ischemic hemisphere of mice (*n* = 8). (**K**) Quantification of brain water content in the ischemic hemisphere for each group (*n* = 6). (**L**) Intracerebral hemorrhage (*n* = 8). Results are expressed as means ± SD. * *p* < 0.05, ** *p* < 0.01, *** *p* < 0.001 versus Model group, ## *p* < 0.01, ### *p* < 0.001 versus Sham group.

We initially conducted the fractionation of DBE using solvents with varying polarities and subsequently evaluated the antithrombotic activity of the resultant fractions utilizing a rat carotid artery thrombosis model induced by FeCl_3_. The selected solvents, including PE, CH_2_Cl_2_, EA, and MeOH, collectively encompassed nearly the entire polarity range pertinent to traditional Chinese medicine (TCM) components. This approach enabled the comprehensive and interference-free separation of the chemical constituents from TCM, attributable to the distinct polarities of these solvents. Thus, the initial separation was carried out using column chromatography, yielding four distinct polar fractions of DBE: Fr. PE, Fr. CH_2_Cl_2_, Fr. EA, and Fr. MeOH (Figure 2A). Subsequently, we assessed the antithrombotic activity of the various polar fractions of DBE by employing an FeCl_3_-induced rat carotid artery thrombosis model (as shown in Figure 2B). Firstly, the monitoring of carotid artery blood flow indicated that thrombosis formation was characterized as a 60% reduction in baseline flow. Following 5 consecutive days of intragastric administration, both the Fr. MeOH (204 mg/kg) and Fr. EA (342 mg/kg) groups exhibited significantly restored blood flow compared to the Model group (Figure 2C). Notably, the Fr. EA group exhibited antithrombotic efficacy comparable to that of the original DBE. Consistent with these findings, the wet thrombosis mass in the Fr. EA and DBE group showed a statistically significant reduction relative to the Model group (Figure 2D). Taken together, these results suggest that Fr. MeOH and Fr. EA are the principal bioactive constituents responsible for mediating the antithrombotic effects of DBE.

### 2.4. Active Fraction in DBE for Alleviating Ischemic Stroke

After confirming the antithrombotic activities of the Fr. MeOH and Fr. EA of DBE, we further investigated their neuroprotective effects using a PT-stroke model (Figure 2E). Mice were subjected to consecutive 3-day intragastric administration. Initial cerebral perfusion assessment using laser speckle imaging revealed a characteristic blood flow pattern (Figure 2F). The baseline cerebral blood flow in the Model group decreased to 40% of that in the Sham group, while treatment with Fr. MeOH (204 mg/kg), Fr. EA (342 mg/kg), and DBE (600 mg/kg) resulted in recovery to approximately 60% of the Sham group’s baseline flow (Figure 2H). Subsequently, a neurological evaluation was conducted using a modified Neurological Severity Score (mNSS), where higher scores were directly correlated with greater neurological impairment. The Model group exhibited the highest mNSS scores, which were significantly attenuated in the Fr. MeOH, Fr. EA, and DBE groups (Figure 2G). Parallel assessments involved the quantification of Evans Blue extravasation and the measurement of cerebral edema via brain water content analysis. The quantification of Evans Blue demonstrated an intact blood–brain barrier (BBB) in the Sham group, in contrast to significant leakage observed in the Model group. Fr. EA and DBE group treatments substantially reduced extravasation (Figure 2I,J), whereas Fr. MeOH failed to mitigate leakage, which was associated with hemorrhagic transformation (HT). Cerebral edema measurements validated these observations, demonstrating a significant increase in brain water content in the Model group relative to the Sham group. However, treatment with Fr. EA counteracted this increase (Figure 2K). Therefore, the administration of Fr. EA was associated with a reduction in BBB permeability in PT-stroke mice, which in turn alleviated cerebral edema. Experiments investigating intracerebral hemorrhage in PT-stroke mice showed that treatment with Fr. MeOH induced HT, whereas treatment with Fr. EA significantly mitigated the risk of HT (Figure 2L). The aforementioned experimental results indicate that Fr. EA alleviates ischemic stroke by healing BBB damage. Conversely, Fr. MeOH exacerbates BBB impairment, thereby contributing to the development of HT.

### 2.5. Evaluation of Compounds in the Fr. EA of DBE for Their Ability to Inhibit ADP-Induced Platelet Aggregation

**Figure 3:**
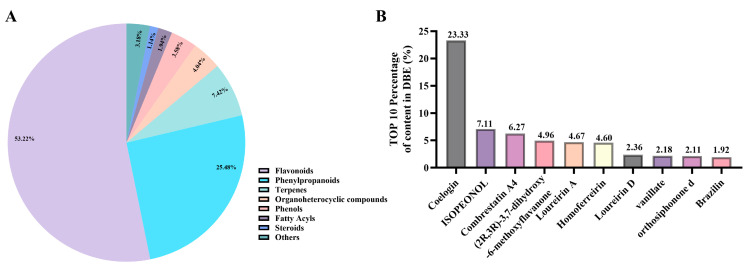
Comprehensive analysis of the chemical composition of DBE. (**A**) Structural classification of compounds included in DBE. (**B**) The top ten most abundant compounds within DBE.

Previous findings have demonstrated that the Fr. EA of DBE significantly inhibits ADP-induced platelet aggregation and confers neuroprotection in ischemic stroke. Therefore, it is essential to further investigate the specific bioactive components within Fr. EA that are responsible for its antiplatelet effects, as well as to explore the underlying mechanisms of its neuroprotective effects. When conducting chemical composition analysis, we began by performing a comprehensive phytochemical analysis in DBE using UHPLC-MS (Thermo, Waltham, MA, USA). The analysis demonstrated that DBE are predominantly composed of flavonoids (53.22%), phenylpropanoids (25.48%), terpenoids (7.42%), organoheterocyclic compounds (4.04%), phenols (3.58%), fatty acid (1.95%), steroids (1.14%), and other components (3.1%). These findings are illustrated in Figure 3A, while Figure 3B showcases the ten most abundant compounds identified within DBE.

**Figure 4:**
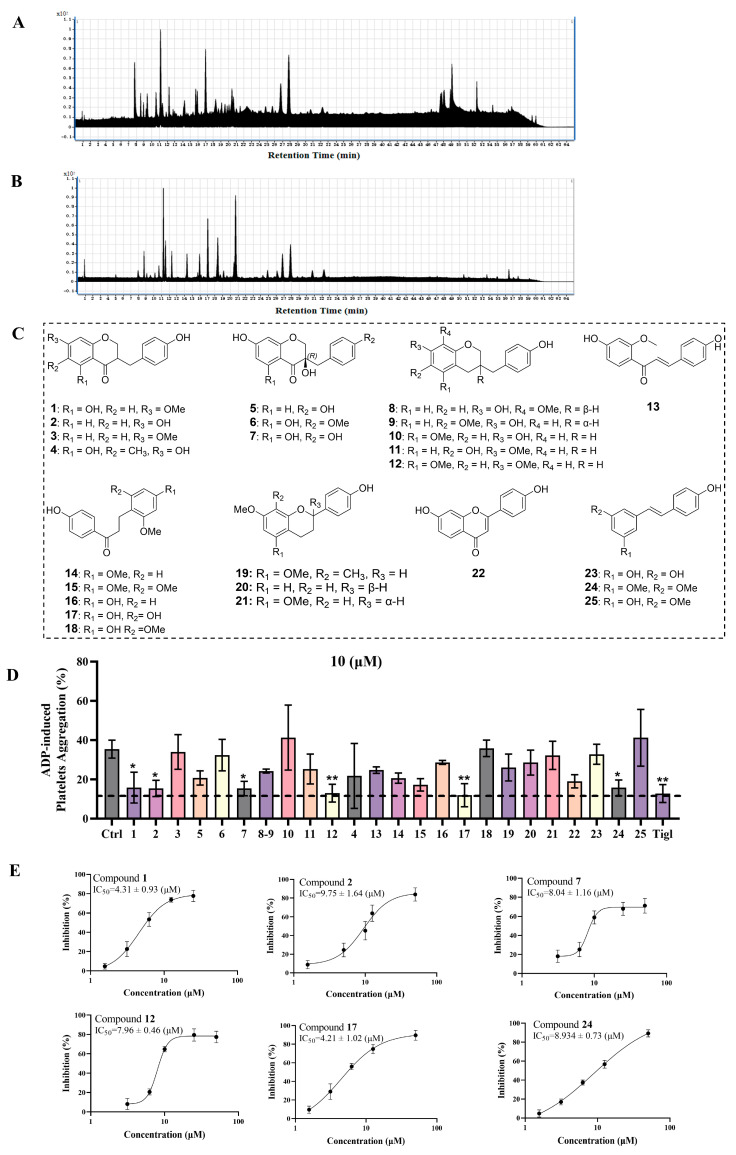
Screening for antiplatelet compounds (ADP-induced) from the Fr. EA of DBE. (**A**) The base peak ion (BPI) chromatogram of Fr. EA derived from DBE in positive ion mode. (**B**) The BPI chromatogram of Fr. EA derived from DBE in negative ion mode. (**C**) Structure representations of the prototype compounds in Fr. EA derived from DBE. (**D**) Preliminary identification of compounds in Fr. EA with antiplatelet platelet aggregation activity at 10 μM (*n* = 3), the dashed line means positive control tigl’s platelets aggregation. (**E**) The 50% inhibitory concentration (IC_50_) values of the active compounds (*n* = 4). Results are expressed as means ± SD. * *p* < 0.05, ** *p* < 0.01 versus Control group.

Based on the preliminary chemical composition analysis results obtained from DBE and considering that the activity of the Fr. EA in DBE was determined to be optimal, we subsequently further isolated and comprehensively characterized its chemical composition. The total ion chromatograms of Fr. EA in both positive and negative ion modes are depicted in Figure 4A,B. Through an integrated approach employing multiple chromatographic techniques, including silica gel chromatography, gel filtration chromatography (Sephadex LH-20), and semi-preparative HPLC, we successfully isolated and identified a total of 25 compounds from Fr. EA (as shown in Figure 4C). The structure of the isolated compounds was determined by comparing their NMR spectroscopic data with those reported in the literature.

These 25 compounds were subjected to an initial in vitro assessment for their antiplatelet activity. The positive control, ticagrelor (10 μM), exhibited a pronounced inhibitory effect on ADP-induced platelet aggregation. In a concurrent screening, six compounds (compounds **1**, **2**, **7**, **12**, **17**, and **24**) demonstrated notable antiplatelet activity by inhibiting platelet aggregation at a concentration of 10 μM (Figure 4D). The spectroscopic data of the six active compounds are shown in Table 1 and Table 2. Preliminary screening for anti-platelet aggregation activity revealed that the six compounds in Fr. EA exhibited significant inhibition of ADP-induced platelet aggregation. Thereafter, the optimal active molecules were further screened from among these active molecules.

Our preliminary screening of the Fr. EA derived from DBE identified six compounds capable of inhibiting ADP-induced aggregation in vitro. To further assess the differences in their inhibitory activities, the half-maximal inhibitory concentration (IC_50_) values were determined. Quantitative analysis revealed that compound **1** (IC_50_ = 4.31 ± 0.93 μM) and compound **17** (IC_50_ = 4.21 ± 1.02 μM) exhibited superior inhibitory effects compared to the other candidates (Figure 4D). The remaining compounds (**2**, **7**, **12**, **24**) showed moderate inhibitory activity, as characterized by their relatively high IC_50_ values. Consequently, the IC_50_-based screening of ADP-induced platelet aggregation suggests that compounds **1** and **17** are likely to be the predominant active molecules within Fr. EA.

### 2.6. Identification of the Most Promising Compounds for Binding to the P2Y12 Receptor

**Figure 5:**
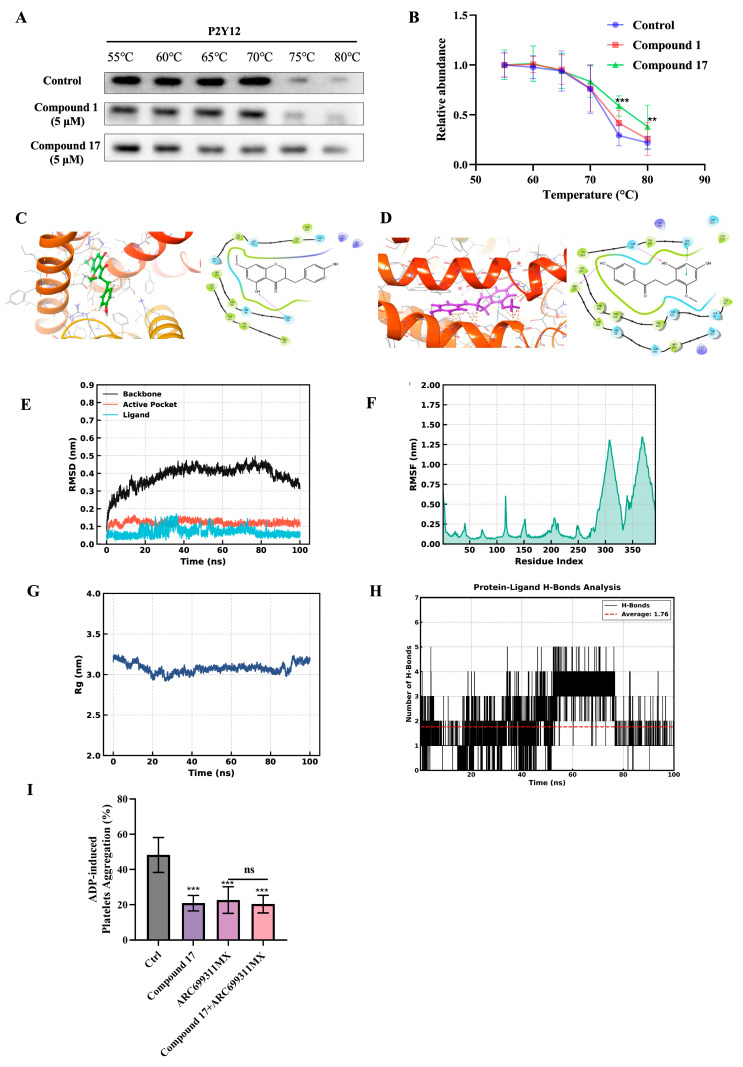
Identification of the most promising compounds for binding to the P2Y12 receptor. (**A**,**B**) The thermostability of compounds **1** and **17** on the P2Y12 protein was confirmed using CETSA. (**C**) Predicted binding modes of compound **1** with the target protein P2Y12. (**D**) Predicted binding modes of compound **17** with the target protein P2Y12. (**E**–**H**) Molecular dynamics simulations at 100 ns: (**E**) root mean square deviation (RMSD) of the protein backbone, active site, and ligand; (**F**) residue root mean square fluctuation (RMSF); (**G**) radius of gyration of the protein; (**H**) number of protein–ligand hydrogen bonds. (**I**) Inhibition of ADP-induced platelet aggregation by the P2Y12 antagonist. Results are expressed as means ± SD. ** *p* < 0.01, *** *p* < 0.001 versus Model group.

Given that ADP binding to the P2Y12 receptor on the platelet surface triggers platelet activation and subsequent aggregation, the most potent compounds identified in our screening, compounds **1** and **17**, were subjected to further assessment of their binding affinities as potential P2Y12 receptor antagonists [23]. The engagement of compounds **1** and **17** with P2Y12 was evaluated using CETSA. Compound **17** demonstrated concentration-dependent stabilization of the P2Y12 thermal denaturation profile within the temperature range of 55–80 °C. In contrast, compound **1** exhibited markedly diminished thermal stabilization efficacy under the same experimental conditions (Figure 5B,C). This thermal stabilization effect was consistent with the binding affinity of compound **17** for P2Y12, as indicated by the reduced protein degradation under thermal stress relative to vehicle controls.

In addition to evaluating the binding affinity using CETSA, we conducted computational docking analyses of compounds **1** (Figure 5C) and **17** (Figure 5D) with the P2Y12 protein using the Schrödinger software (2022). For compound **17**, the docking results yielded an XP Glide Score of −8.273 (more negative than compound **1**’s −7.232, indicating stronger binding affinity), a Glide model of −59.402 (more negative than compound **1**’s −47.764, reflecting better binding stability), and an XP Pose Rank of 1. In terms of the binding mode, compound **17** formed more extensive hydrogen bonds (e.g., with Met 152, Tyr 109) and engaged in hydrophobic interactions with a broader range of residues (including Tyr 105, Tyr109, Phe106), along with potential polar interactions. These synergistic and abundant intermolecular interactions demonstrated that compound **17** exhibits superior binding affinity and stability compared to compound **1**.

In our prior comprehensive compositional analysis of DBE, Loureirin D (compound **17**) was identified as the predominant component, comprising 2.36% (*w*/*w*) of the total composition (Figure 3B). Subsequently, we further examined the in vivo metabolic components of Fr. EA and successfully detected Loureirin D (compound **17**) in the serum of mice treated with Fr. EA (Table 3).

Based on the aforementioned experimental results, we concentrated our efforts on compound **17** for further evaluation of its in vitro ADP-induced antiplatelet activity (Figure 5E). The results indicate that compound **17** exhibited a dose-dependent increase in anti-platelet aggregation activity at concentrations ranging from 6 to 24 mg/kg.

The computer docking predictions and CETSA experiments further evidenced that compound **17** exhibits binding affinity toward the P2Y12 receptor. ADP is well-established as a ligand for the P2Y12 receptor, which is crucial in initiating platelet activation through G-protein coupling, ultimately leading to thrombus formation. Therefore, it can be hypothesized that compound **17** exerts its antiplatelet aggregation activity by inhibiting the interaction between ADP and P2Y12. Figure 5E (RMSD) illustrates that the protein backbone stabilizes at approximately 0.4 nm after 20 ns, while the active site and ligand exhibit low RMSD values (<0.1 nm), indicating stable ligand binding without significant conformational drift. Figure 5F (RMSF) reveals that most residues displayed minimal flexibility (RMSF < 0.5 nm), with elevated fluctuations observed only in terminal loops—regions that do not compromise the structural rigidity of the active site. Figure 5G (radius of gyration) indicates that the protein remains a compact conformation (Rg: 3.0–3.2 nm), indicating no risk of unfolding. Figure 5H (protein–ligand H-bonds) shows that an average of 1.76 hydrogen bonds were dynamically maintained throughout the simulation, providing stable intermolecular interactions. To further validate the P2Y12-targeted effect of compound **17**, platelet aggregation assays were conducted using the well-established antagonist ARC69931MX. Both compounds individually induced significant inhibition of ADP-induced aggregation. However, when platelets were pre-incubated with ARC69931MX to saturate the receptor, the subsequent addition of compound **17** resulted in no additional inhibitory effect. Collectively, these findings suggest that compound **17** achieves its antithrombotic effects via binding to the P2Y12 receptor, thereby suppressing its activation.

### 2.7. Assessment of the Anti-Ischemic Stroke Efficacy of Compound 17 In Vivo

**Figure 6:**
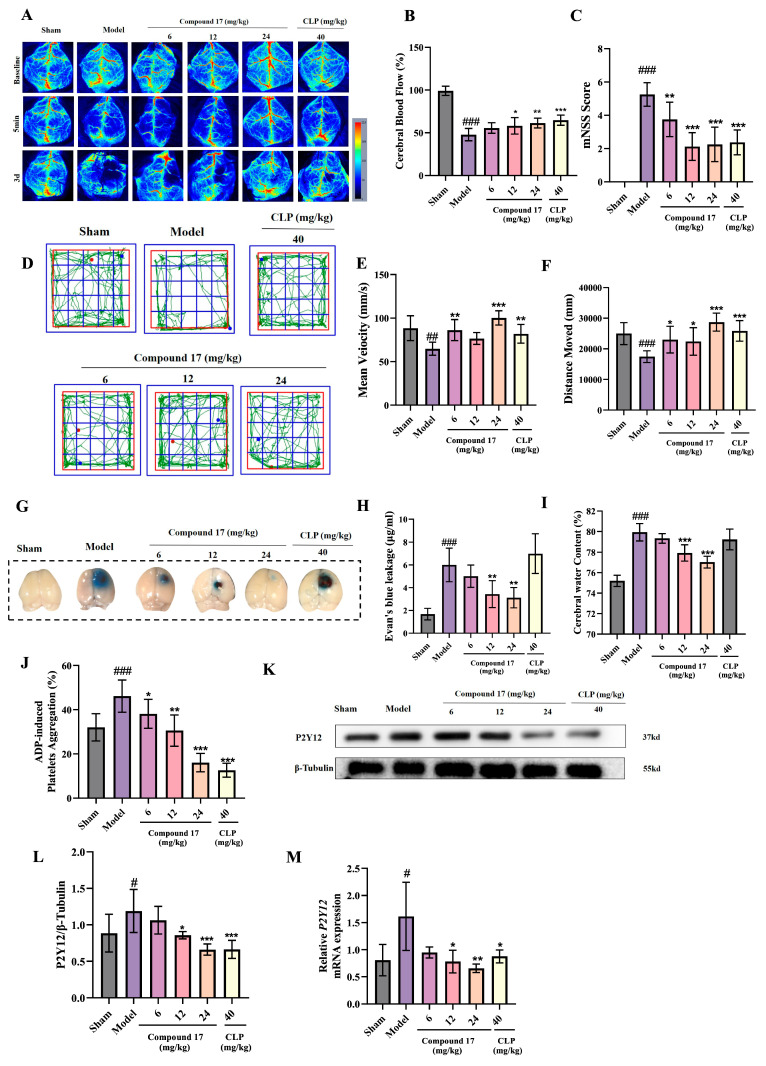
The antithrombotic and stroke-protective effects of compound **17**. (**A**) Representative images of CBF at 3 d in every group. (**B**) Statistical analysis of CBF for B (*n* = 8). (**C**) Neurological function score in mice (*n* = 8). (**D**) Representative motion trajectory of the open-field test, red dot means the starting trail and blue dot means the ending trail. (**E**) The statistical analysis of distance moved and mean velocity (*n* = 8). (**F**) Statistical analysis of distance moved and mean velocity (*n* = 8). (**G**) Representative Evans Blue staining images. (**H**) Quantitative analysis of Evans Blue extravasation in the ipsilateral ischemic hemisphere of mice (*n* = 6). (**I**) Quantification of brain water content in the ischemic hemisphere across experimental groups (*n* = 6). (**J**) Quantification of ADP-induced platelet aggregation (*n* = 8). (**K**) Analysis of the protein level of P2Y12 in brain tissue (*n* = 6). (**L**) Quantification of data presented in (**K**). (**M**) RT-qPCR analysis of P2Y12 mRNA levels in brain tissue. Results are expressed as means ± SD. * *p* < 0.05, ** *p* < 0.01, *** *p* < 0.001 versus Model group, # *p* < 0.05, ## *p* < 0.01, ### *p* < 0.001 versus Sham group.

After confirming the antithrombotic activity and inhibition of platelet aggregation of compound **17**, its efficacy was further assessed in the PT-stroke model. The experimental results demonstrated that compound **17**, when administered via gavage for 3 days, significantly enhanced cerebral blood flow in a dose-dependent manner compared to the Model group (Figure 6A,B). At 3 days of treatment, the mNSS and open-field tests revealed that the high-dose group (24 mg/kg) experienced not only ameliorated neurological deficits (Figure 6C) but also improved motor function in stroke-induced mice, as evidenced by increases in mean velocity and total distance traveled (Figure 6D–F). The positive control CLP (40 mg/kg) demonstrated comparable therapeutic efficacy. Evans Blue extravasation assays and cerebral water content measurements further demonstrated the dose-dependent preservation of BBB integrity by compound **17**, with a significant reduction in thrombus-induced dye leakage across treatment groups (Figure 6G–I). Based on the aforementioned experiments, it was concluded that compound **17** alleviates ischemic stroke injury by attenuating BBB disruption caused by cerebral thrombosis and ameliorating motor dysfunction in PT-stroke mice. We further verified the effect of compound **17** on platelet aggregation in PT-stroke mice after in vivo administration (Figure 6J). The results indicate that compound **17** could significantly inhibit platelet aggregation in PT stroke mice. To assess P2Y12 expression in vivo, Western blot analysis was performed. Compared with the Model group, which exhibited upregulated P2Y12 receptor expression, treatment with compound **17** significantly reduced P2Y12 levels in brain tissue (Figure 6K,L). Complementary qPCR analysis of cortical tissue confirmed the consistent downregulation of P2Y12 mRNA (Figure 6M).

## 3. Discussion

Ischemic stroke, defined by cerebral hypoperfusion resulting from thromboembolic vascular occlusion and subsequent neuronal ischemia–reperfusion injury, poses a significant therapeutic challenge due to its intricate pathophysiological mechanisms and the hemorrhagic risks associated with conventional thrombolytic therapies [2]. Thrombotic events, primarily driven by pathological platelet activation, represent the predominant etiological factor in ischemic stroke [24]. The long-term use of antiplatelet drugs currently in use, such as aspirin and clopidogrel, may lead to the risk of hemorrhagic complications and thrombocytopenia. DBE exhibit pharmacological effects in treating cardiovascular diseases. We conducted a systematic evaluation of the antithrombotic efficacy of DBE and explored the specific antiplatelet compounds. The research establishes a mechanistic foundation for developing novel antithrombotic therapeutics with enhanced efficacy and safety profiles, potentially advancing arterial thrombosis treatment paradigms.

Consequently, in this study, we employed the FeCl_3_-induced carotid artery thrombosis animal model and the PT-stroke thrombosis model to systematically evaluate and screen the therapeutic efficacy of DBE on ischemic stroke through antithrombotic activity.

We demonstrated that DBE enhances cerebral blood flow in mice with thrombotic stroke and markedly suppresses platelet aggregation induced by ADP. Furthermore, histological analyses via HE staining and TUNEL staining of brain tissue sections revealed that DBE can alleviate hypoglycemic and hypoxic damage in brain tissue, thereby confirming its efficacy in treating thrombotic stroke.

Through bioactivity-guided fractionation combining FeCl_3_-induced thrombosis screening and PT-stroke validation, Fr. EA was identified as the primary bioactive component, exhibiting stronger antiplatelet activity (ADP-induced) compared to other fractions. Furthermore, the compounds in Fr. EA were isolated and identified using chromatography and semi-preparative high-performance liquid chromatography (HPLC). Subsequently, the antiplatelet aggregation activity (ADP-induced) of the molecules in Fr. EA was screened. It was determined that compounds **1** and **17** are potential active molecules in DBE. Based on the pivotal role of ADP–P2Y12 interaction in initiating platelet aggregation, binding affinity assessments of the lead compounds **1** and **17** targeting this receptor were conducted through CETSA. The CETSA results confirmed the target engagement of compound **17** with P2Y12. Molecular docking studies revealed that compound **17** forms a greater number of hydrogen bonds and additionally engages in π–π stacking interactions with P2Y12 compared to compound **1**. Furthermore, compound **17** was identified as one of the top ten abundant compounds in the chemical profiling of DBE by UHPLC-MS and was also detected in the blood components of Fr. EA. Further in vitro experiments confirmed that compound **17** exhibited a dose-dependent inhibitory effect on ADP-induced platelet aggregation. In PT-stroke models, compound **17** was shown to enhance cerebral blood flow and improve neurological function scores. Meanwhile, compound **17** reduced Evans Blue extravasation, suggesting that it may reduce the risk of hemorrhagic transformation during thrombolytic therapy. In subsequent studies, we will further investigate the mechanism by which it attenuates hemorrhagic transformation through the protection of the blood–brain barrier, thereby exerting a robust neuroprotective effect against ischemic injury. Furthermore, we assessed the levels of P2Y12 in brain tissue and observed downregulation at both the protein and mRNA levels. This decrease may represent a feedback response to prolonged receptor antagonism. The precise biological implications of this finding warrant further investigation.

The P2Y12 receptor functions as the principal ADP-responsive purinergic receptor on platelet membranes, and its activation initiates critical thrombotic signaling cascades [25]. Upon ADP binding, this G protein-coupled receptor triggers platelet activation through intracellular calcium mobilization and dense granule secretion. The released ADP establishes an autocrine amplification loop via P2Y12 engagement, thereby reinforcing thrombus stability through the coordinated activation of secondary signaling pathways, including TXA2 synthesis and αIIbβ3 integrin activation [26]. This self-perpetuating mechanism underpins the therapeutic rationale for P2Y12 antagonists, such as clopidogrel and ticagrelor, in the management of arterial thrombosis. Clinical evidence has consistently demonstrated their capacity in attenuating both thrombotic propagation [27] and platelet-mediated inflammatory responses [28]. Beyond its canonical role in platelet activation, the P2Y12 receptor demonstrates broad physiological relevance, with functional expression observed across multiple tissue systems [29]. In vascular smooth muscle cells, this receptor plays a critical role in pathways governing phenotypic plasticity and vascular remodeling processes, contributing to pathological conditions such as atherosclerotic progression and post-angioplasty restenosis [30]. Within the neurovascular unit, microglial P2Y12 activation orchestrates chemotactic responses and modulates neuroinflammatory cascades through dynamic interactions with ischemic penumbra microenvironments [31].

**Figure 7:**
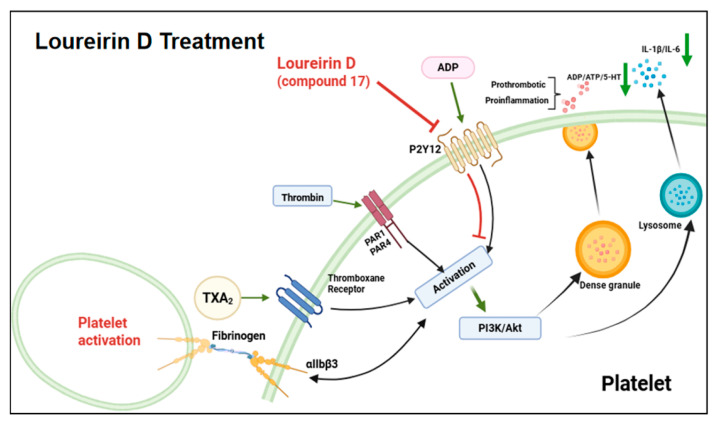
Schematic representation of the mechanism of action of Loureirin D in the treatment of thrombosis in rats.

Our investigation demonstrated that DBE significantly suppresses ADP-induced platelet aggregation. Through bioactivity-guided screening, compounds **1** and **17** were identified as potent inhibitors of ADP-mediated platelet activation. Molecular docking simulations combined with CETSA analysis revealed a significantly enhanced binding affinity of compound **17** towards the P2Y12 receptor, indicating its potential role as a P2Y12 receptor antagonist. Crucially, to investigate whether the effect of Loureirin D is mediated via the P2Y12 receptor, we performed a platelet aggregation assay. When platelets were pre-incubated with a saturating concentration of the well-established P2Y12 antagonist ARC69931MX, the subsequent inhibitory effect of compound **17** was significantly attenuated. Furthermore, subsequent in vivo validation using cerebral thrombosis models confirmed the therapeutic efficacy of compound **17**, which was associated with a marked downregulation of P2Y12 expression in the cerebral vasculature. This modulation of receptor expression may represent a compensatory mechanism underlying prolonged in vivo inhibition of P2Y12. However, the precise pathways involved remain unclear and warrant further investigation. Compound **17** exerts its antithrombotic activity through antagonism of the ADP–P2Y12 receptor interaction, thereby effectively suppressing platelet activation and adhesion processes that are critical for thrombus formation (Figure 7). These findings establish compound **17** as the principal bioactive component of DBE responsible for its therapeutic efficacy against cerebral thrombosis, offering valuable mechanistic insights into its pharmacological targets and mode of action. In conclusion, Loureirin D (compound **17**) can treat thrombosis by inhibiting the activation of platelets by suppressing the P2Y12 receptor, thereby reducing the release of prothrombotic factors such as ADP. The discovery of P2Y12 inhibitors from natural sources has been limited, with few reported instances such as notoginsenoside Ft1 isolated from Panax notoginseng [32] and (−)-epigallocatechin gallate (EGCG) from green tea [33]. Compared with these compounds, Loureirin D demonstrated a comparable or even superior inhibitory activity (IC_50_ = 4.21 ± 1.02 μM). Furthermore, in vivo studies demonstrated that Loureirin D also exerts a protective effect on the BBB, indicating that it possesses both antithrombotic activity and neuroprotective properties.

However, the current study has several limitations that warrant acknowledgment. Although this study identified P2Y12 antagonism as a key mechanism, the exclusivity of this pathway requires validation using genetically modified models. Future studies should aim to delineate potential off-target effects and investigate the cross-talk with complementary thrombotic signaling pathways.

## 4. Materials and Methods

### 4.1. General Experimental Procedures

NMR data were acquired using Bruker AM-400 spectrometers (Rheinstetten, Germany) with tetramethylsilane (TMS) as the internal standard. Semi-preparative HPLC was performed on either an Agilent 1200 system (Santa Clara, CA, USA) or a WUFENG HPLC system (Wuhan, China), equipped with a reversed-phase (RP) C18 column (5 μm, 10 × 250 mm, pronaos-C18; or 10 μm, 21.2 × 250 mm, pronaos-C18). CC was carried out using silica gel (40–80, 200–300, and 300–400 mesh; Qingdao Marine Chemical Inc., Qingdao, China), and Sephadex LH-20 (Adamas life BR, Shanghai, China) for separation and purification purposes.

### 4.2. Plant Materials

The dried Dragon’s Blood phenolic extracts were provided by Jiangsu Kanion Pharmaceutical Co., Ltd. (Lianyungang, China). in July 2024. A voucher sample (No. WP20240715) has been deposited in the herbarium of the Innovative Research Institute of Traditional Chinese Medicine at SHTCM.

### 4.3. Isolation and Purification

DBE (25.0 g) were subjected to silica gel column chromatography and eluted with a gradient of petroleum ether-dichloromethane-ethyl acetate-methanol, yielding four major fractions (Fr. PE, Fr. CH_2_Cl_2_, Fr. EA, and Fr. MeOH). Subsequently, fraction Fr. EA (14.2 g) was further separated on a silica gel column using a CH_2_Cl_2_-EtOAc gradient (from 95:5 to 0:1), resulting in nine sub-fractions (A–I). Fraction B (1.0 g) was purified via Sephadex LH-20 column chromatography (MeOH) to produce two sub-fractions (B1–B2). Further purification of B1 was achieved using semi-preparative HPLC (MeOH/H_2_O, 69:31), leading to the isolation of compounds **20** (10.0 mg, t_R_ = 23 min), **21** (11.0 mg, t_R_ = 27 min), and **24** (9.0 mg, t_R_ = 21 min). Compounds **12** (12.0 mg, t_R_ = 32 min) and **19** (3.0 mg, t_R_ = 32 min) were obtained from B2 under identical HPLC conditions as those used for B1. D (4.0 g) was applied to Sephadex LH-20 column chromatography (eluted with MeOH) to yield a fraction (D2) and compound **25** (12.0 mg). Fraction D2 was further separated using repeated silica gel column chromatography (CH_2_Cl_2_/EtOAc, gradient elution from 96:2 to 80:20, then 1:1, *v*/*v*), resulting in five subfractions (D2a–D2e). These subfractions were subsequently purified using semi-preparative HPLC. Specifically, compounds **14** (6.0 mg, t_R_ = 45 min) and **15** (7.0 mg, t_R_ = 47 min) were obtained from D2a using ACN/H_2_O (38:62) as the mobile phase. Compound **3** (3.0 mg, t_R_ = 38 min) was isolated from D2b under the same conditions. Compound **6** (6.0 mg, t_R_ = 24 min) was obtained from D2c. A pair of mixtures, compounds **8** (7.0 mg, t_R_ = 24 min) and **9** (3.0 mg, t_R_ = 24 min) from D2d, were identified. Finally, from D2e, compounds **4** (4.0 mg, t_R_ = 46 min), **10** (10.0 mg, t_R_ = 30 min), and **11** (7.0 mg, t_R_ = 26 min) were purified using semi-preparative HPLC (MeOH/H_2_O, 54:46) as the mobile phase. Additionally, compounds **7** (3.0 mg, t_R_ = 25 min) and **17** (10.0 mg, t_R_ = 28 min) were separated from fraction D1 using semi-preparative HPLC with ACN/H_2_O (25:75) as the mobile phase. F (1.0 g) was subjected to semi-preparative HPLC separation (ACN/H_2_O, 28:72) to yield three compounds: **2** (32.0 mg, t_R_ = 22 min), **16** (21.0 mg, t_R_ = 27 min), and **18** (19.0 mg, t_R_ = 36 min). Three fractions (G1-G3) were obtained by repeatedly eluting G using silica gel column chromatography (CH_2_Cl_2_/MeOH, 99:1-95:5, *v*/*v*) followed by Sephadex LH-20 (MeOH). Compound **23** (40.0 mg) was isolated from fraction G3, while compounds **5** (6.0 mg, t_R_ = 18 min, ACN/H_2_O, 25:75) and **13** (7.0 mg, t_R_ = 41 min, ACN/H_2_O, 30:70) were purified via semi-preparative HPLC. Two fractions (**I1**–**I2**) were purified using Sephadex LH-20 chromatography with MeOH as the eluent. Compound **1** (80.0 mg), obtained via dichloromethane crystallization, was isolated from **I1**, while compound **22** (7.0 mg), obtained through ethyl acetate crystallization, was isolated from **I2**.

Structure Elucidation. A total of 25 known compounds (**1**–**25**, Figure 3) were identified based on the comparison of their NMR data with those reported in the literature. These compounds include: 5-hydroxy-7-methoxy-3-(4′-hydroxybenzyl)-4-chromanone (**1**) [34]; 7, 4′-dihydrohomoisoflavanone (**2**) [35]; 3-(4-hydroxybenzyl)-7-methoxychroman-4-one (**3**) [36]; 5, 7-dihydroxy-6-methyl-3-(4′-hydroxy-benzyl)-chroman-4-one (**4**) [37]; 3′-deoxysappanone B (**5**) [38]; eucomol (**6**) [39]; loureiriol (**7**) [39]; 7-hydroxy-3-(4-hydroxybenzyl)-8-methoxychroman (**8**) [40]; 6-hydroxy-7-methoxy-3-(4′-hydroxybenzyl) chromane (**9**) [41]; 7, 4′-dihydroxy-5-methoxyhomoisoflavane (**10**) [40]; 7, 4′-homoisoflavane (**11**) [35]; 3-(4-hydroxybenzyl)-5, 7-dimethoxychroman (**12**) [42]; 3-deoxysappanchalcone (**13**) [43]; Loureirin A (**14**) [44]; Loureirin B (**15**) [45]; Loureirin C (**16**) [35]; Loureirin D (**17**) [46]; 4, 4′-dihydroxy-2, 6-dimethoxydihydrochalcone (**18**); 4′-hydroxy-5, 7-dimethoxy-8-methylflavan (**19**) [47]; (2*S*)-4′-hydroxy-7-methoxyflavan (**20**) [48]; (2*R*)-caesalflavan B (**21**) [49]; 7, 4′-dihydroxyflavone (**22**) [35]; resveratrol (**23**) [50]; pterostilbene (**24**) [51]; and 3-methoxyresveratrol (**25**) [52].

### 4.4. Animals

Adult male Sprague Dawley rats (weighing 260–280 g) and adult male mice (weighing 20–25 g) were used in this study. The animals were procured from Beijing Weitong Lihua Laboratory Animal Technology Co., Ltd. (Beijing, China). Male subjects were selected based on their stable androgen profile, which is not influenced by the cyclical hormonal fluctuations observed in female rodents, thus reducing experimental variability. All animal procedures were formally approved by the Animal Ethics Committee of Shanghai University of Traditional Chinese Medicine. Animal care and experimental protocols complied with the Institutional Guidelines (Approval number: PZSHUTCM2506260001 and PZSHUTCM2506260002) and were conducted in strict accordance with the NIH Guide for the Care and Use of Laboratory Animals and ARRIVE 2.0 guidelines. Animals were acclimatized in standardized housing facilities, where the ambient temperature was maintained at 22–26 °C and relative humidity at 40–70%. They were housed under controlled photoperiod conditions (12-h light–dark cycle). During the entire experimental period, standard rodent chow and filtered water were provided ad libitum.

### 4.5. Photothrombotic-Induced Stroke (PT-Stroke) Model

Mice were anesthetized using Zoletil 50 (Virbac, Viriat, France) for maintenance. A photothrombotic stroke (PT-stroke) was induced in the right hemisphere to unilaterally lesion the sensorimotor cortex, as previously described [53]. In brief, animals were secured in a stereotactic frame, and the surgical area was sterilized before exposing the skull via a midline skin incision. A cold light source (CHAONAI, 100 mW, 540 nm) was positioned over the right forebrain cortex (anterior/posterior: ±1.5 mm; medial/lateral: 0–2 mm relative to Bregma). Five minutes prior to illumination, Rose Bengal (50 mg/kg in 0.9% NaCl, Sigma, St. Louis, MO, USA) was administered via tail vein injection. Subsequently, the region of interest was illuminated through the intact skull for 5 min. The welfare of the rats was monitored daily throughout the experiment.

### 4.6. Ferric Chloride (FeCl_3_)-Induced Carotid Artery Thrombosis Model

In the cerebral thrombus model, as previously described, FeCl_3_ stimulation of the common carotid artery was performed, followed by mechanical detachment of the thrombus. Rats were initially anesthetized with 3% isoflurane and subsequently maintained under anesthesia with 2.5% isoflurane. Following disinfection of the surgical site, a midline cervical incision was made to expose the right common carotid artery under microscopic guidance. A filter paper saturated with a 40% FeCl_3_ solution was then applied to the right common carotid artery for 5 min. In the Sham group, the same operation was conducted except that the filter paper was soaked in saline instead of FeCl_3_. The welfare of all rats was monitored daily throughout the duration of the experiment.

### 4.7. Quantification of Blood Flow

Carotid blood flow in rats was measured using laser Doppler flowmetry both before and after surgery. The laser probe was positioned at the ischemic core of the carotid artery. Following a 5-min stabilization period, the blood flow index was recorded. The assessment of blood flow change was performed by comparing the blood flow measurements obtained 5 days post-administration with the baseline values.

Cerebral blood flow was measured by laser Doppler flowmetry. A surgical incision was made in the scalp to expose the skull. The computer-controlled optical scanner was positioned 15 cm directly above the skull, and a red laser beam was projected onto the surface of the skull. After configuring the relevant parameters, the autofocus function was employed to acquire high-resolution images, and the cerebral blood flow data at various time points were collected. The moorFLPI Review software (1.0) was subsequently utilized to analyze the cerebral blood flow data.

### 4.8. Hematoxylin and Eosin (H&E) Staining

Cerebral sections were subjected to standardized histological processing, which included xylene dewaxing and ethanol gradient dehydration. This was followed by hematoxylin and eosin (H&E) histochemical staining. Morphological assessment was performed using a confocal laser-scanning microscope (Nikon, Tokyo, Japan) for imaging acquisition.

### 4.9. TUNEL Staining

Mouse brains were fixed with 4% paraformaldehyde solution and subjected to gradient dehydration. The sections were then equilibrated to room temperature (18–25 °C). Subsequently, they were incubated with a solution containing 0.2% H_2_O_2_ and methanol for 0.5 h to prevent endogenous peroxidase activity. Following this step, the sections were treated with the TUNEL reaction mixture (Roche, Basel, Switzerland) at 37 °C for 1 h. Apoptotic cells were visualized using an inverted laser scanning confocal microscope (Nikon, Tokyo, Japan), and quantification of apoptotic cells was performed with ImageJ software version 1.50i.

### 4.10. Neurological Score

At 3 days post-administration, the neurological function of the mice in each group was assessed using modified neurological severity scores (mNSS) as outlined in the prior literature [54].

### 4.11. Open Field Test

The open field test (OFT) was performed in accordance with established protocols, incorporating standardized modifications as described. The testing apparatus consisted of a 50 × 50 × 35 cm open-field arena, which was virtually divided into a 25-grid configuration. The central quadrant (25 × 25 cm) was designated as the anxiety-sensitive zone, while the peripheral areas were identified as exploration zones. Following a 30-min acclimatization period in the testing room under controlled illumination (50 lux), individual mice were placed at the center of the arena and permitted to explore freely for 300 s. Locomotor parameters, including ambulatory distance (mm) and velocity (mm/s), were quantified using automated tracking software (XR-VT 2.0) under standardized conditions (noise-free environment, constant ambient light). Inter-trial sanitation was systematically performed using 75% ethanol to eliminate olfactory cues between subjects.

### 4.12. Evans Blue Leakage Assay

The permeability of the BBB was assessed using Evans Blue (EB) dye (Sigma, St. Louis, MO, USA). Mice were intravenously administered 2% EB through via tail vein injection at a dose of 2 mL/kg, 90 min prior to euthanasia. To eliminate intravascular dye contamination, mice were perfused with cold 0.9% saline before brain samples were collected for subsequent imaging analysis. Brain tissues was then dissected, weighed, and homogenized in 50% trichloroacetic acid at a ratio of 1:3 (*w*/*v*). Following homogenization, samples were centrifuged at 12,000× *g* for 30 min. The resulting supernatant was used for the EB colorimetric assay. Absorbance measurements of the supernatant were performed at 620 nm using the Thermo Fisher Multiskan FC microplate reader (Waltham, MA, USA).

### 4.13. Cerebral Water Content Analysis

Mice were euthanized, and their brains were quickly excised. The wet weight of the right cerebral hemisphere was measured and recorded. Subsequently, the hemispheres were dried in an oven at 60 °C for 3 days, after which the dry weight was measured and documented. The percentage of cerebral water content was calculated using the formula: (wet weight − dry weight)/wet weight × 100%.

### 4.14. Assessment of Cerebral Hemoglobin Levels

Cerebral tissue processing was performed subsequent to trans-cardiac perfusion with saline in murine subjects. Upon completion, excised brains were promptly cryopreserved at −80 °C. Ischemic tissue homogenization was carried out in 2% EDTA solution, followed by centrifugation (12,000 rpm, 15 min, 4 °C) to isolate cellular fractions. Hemoglobin quantification was achieved via colorimetric analysis using commercially available assay kits, according to the manufacturer’s instructions.

### 4.15. Preparation of Rat Platelets

Blood samples were collected from Sprague Dawley (SD) rats through abdominal aortic puncture and immediately transferred into acid citrate dextrose (ACD)-containing tubes for anticoagulation. Platelet-rich plasma (PRP) was obtained by centrifuging the blood at 800 rpm for 10 min. Subsequently, platelet-poor plasma (PPP) was prepared by further centrifugation of PRP at 3000 rpm for 10 min and used as the blank control. To isolate the PPP, platelet concentration was adjusted to 4.8 × 10^8^ platelet/mL prior to the aggregation test.

### 4.16. Platelet Aggregation Assay

Platelet aggregation responses to agonist stimulation were assessed using an AggRAM analyzer (Helena Laboratories, Beaumont, TX, USA). After calibration of the instrument, platelet samples were pre-incubated with test compounds at various concentrations or with the control vehicle under static conditions (37 °C for 5 min). Subsequently, specific agonists, including adenosine diphosphate (ADP, 3 μM), arachidonic acid (AA, 1 mM), and thrombin (1 U/mL) were added at predetermined concentrations to induce platelet aggregation. The concentration of each agonist was selected based on preliminary experiments to elicit maximal activation.

### 4.17. Molecular Docking Analysis

The three-dimensional structures of compounds **1** and **17** were generated and energy-minimized using LigPrep in Schrödinger Suite (2022), while the crystal structure of P2Y12 (PDB ID: 4NTJ) was retrieved from the Protein Data Bank (PDB) database and optimized via Schrödinger’s Protein Preparation Wizard (involving protein cleaning, removal of water molecules, and energy minimization with the OPLS4 force field). Compounds **1** and **17** were further preprocessed (including charge assignment, tautomer generation, and conformer enumeration) using LigPrep to ensure compatibility with subsequent docking simulations. The potential active pocket of P2Y12 was predicted using Schrödinger’s SiteMap module (with a calculation threshold set to 0.8–1.2, a range commonly used for druggability evaluation), and the binding site with a SiteMap score closest to 1.0 was selected as the target active pocket, around which a binding site sphere was constructed with a radius of 8.0 Å and coordinates (x = 11.77, y = −3.5, z = 52.3) to fully cover the pocket. Docking simulations were performed using Ligand Docking (2022.1.141) (based on the Glide protocol in Schrödinger) with the OPLS4 force field following a two-step strategy: first, the Glide Standard Precision (SP) mode was used for rapid preliminary docking to screen out energetically favorable conformations of the compounds. The top-ranked conformers from the SP docking were further subjected to the Glide Extra Precision (XP) mode (a core component of Ligand Docking) for high-precision refinement, where XP docking employed rigorous conformational sampling, detailed interaction evaluation, and multi-dimensional scoring. This included Glide Emodel (a weighted energy term combining binding free energy and conformational penalty, with a screening standard of ≤−50 kcal/mol to ensure binding stability), pose quality (assessed by spatial fitting with the active pocket and absence of steric clashes), and docking score (Glide Score) (a composite score integrating intermolecular interactions and solvation effects). The best-docked pose of each compound was selected through comprehensive evaluation of these three XP metrics (prioritizing both binding energy and structural compatibility), followed by visualization of 3D receptor–ligand complex structures to evaluate binding orientation and spatial matching, and generation of 2D interaction diagrams via Schrödinger Maestro to identify key intermolecular interactions.

### 4.18. Molecular Dynamics Simulations and Trajectory Analysis

System preparation and parameterization molecular dynamics (MD) simulations were performed using the AMBER 25 software package. The initial coordinates of the protein–ligand complex were derived from the molecular docking results. To ensure structural integrity, hydrogen atoms were initially stripped and subsequently reconstructed according to the standard geometry parameters of the AMBER ff14SB force field via the pdb4amber module. The partial atomic charges of the ligand were calculated using the AM1-BCC method implemented in the Antechamber module, and the ligand parameters were assigned with the Generalized Amber Force Field 2 (GAFF2). The complex was solvated in a truncated octahedron box using the TIP3P water model, with a minimum buffer distance of 12.0 Å between the solute atoms and the box edges. The system was neutralized by adding counterions (Na^+^/Cl^−^) to mimic physiological conditions. To eliminate steric clashes and geometric irregularities, the system underwent a rigorous five-stage energy minimization protocol utilizing the pmemd.cuda engine. To prevent numerical instability, the steepest descent algorithm was employed throughout the energy minimization process. The minimization sequence proceeded as follows: (1) hydrogen atoms only; (2) solvent molecules and ions; (3) amino acid side chains; (4) protein backbone with weak restraints; and (5) the entire system without restraints. Following minimization, the system was gradually heated from 0 K to 310 K over a period of 50 ps under the NVT ensemble using a Langevin thermostat with a collision frequency (γ) of 5.0 ps^−1^ while weak harmonic restraints (5.0 kcal/mol·Å^2^) were applied to the solute. The SHAKE algorithm was enabled to constrain the bonds involving hydrogen atoms, allowing for a time step of 1 fs during the heating phase. Subsequently, density equilibration was performed under the NPT ensemble (310 K, 1 atm) over a period of 50 ps. The system was then subjected to six stage equilibration, during which the positional restraints on the solute were gradually reduced from 5.0 to 0.0 kcal/mol·Å^2^, and the time step was incrementally increased to 2.0 fs. Finally, a 100 ns unconstrained production simulation was conducted under the NPT ensemble at 310 K and 1 atm. The temperature was maintained using a Langevin thermostat (γ = 2.0 ps^−1^), and the pressure was controlled using the Berendsen barostat. Coordinates were saved every 10 ps for subsequent analysis. Trajectory analysis post-processing and trajectory analysis were performed using the Cpptraj module in AMBER 25. The trajectories were first auto-imaged to correct for periodic boundary conditions and stripped of solvent molecules. The stability and conformational changes in the system were evaluated by calculating the root mean square deviation (RMSD) of the protein backbone, the ligand, and the active site residues (defined as residues within 5 Å of the ligand). The compactness of the complex was assessed using the radius of gyration (Rg). Residue-level flexibility was characterized by the root mean square fluctuation (RMSF) of the Cα atoms. To investigate the binding interactions, hydrogen bond analysis was performed to identify stable contacts between the ligand and the protein, with a distance cutoff of 3.0 Å and an angle cutoff of 135°.

### 4.19. Cellular Thermal Shift Assay (CETSA)

Brain tissues were lysed in RIPA lysis buffer supplemented with phosphatase inhibitors and protease inhibitors. The resulting supernatant obtained after centrifugation was transferred to new 1.5 mL EP tubes for BCA protein quantification analysis. It was then incubated with either 5 μM of compound **1** or compound **17** or an equivalent volume of DMSO at 4 °C for 3 h. Subsequently, the tissue lysates in each tube were heated to designated temperatures (55–80 °C) for 3 min, followed by equilibration at ambient temperature (25 ± 1 °C) for 3 min. Afterward, the samples were maintained at room temperature for an additional 3 min. Finally, the soluble fractions were collected and subjected to Western blot analysis. Immunoblots were semi-quantitatively analyzed using ImageJ software (version 1.52a), and the resulting data were normalized. Subsequently, the thermal stability curves were fitted using nonlinear regression.

### 4.20. Real-Time Quantitative PCR (RT-qPCR)

The total mRNA was extracted from the brain tissue using Trizol reagent (Vazyme, Nanjing, China, Catalog No. R401-01). The concentration of the extracted mRNA was quantified using an ultramicro spectrophotometer (DeNovix, Wilmington, DE, USA, Model TFB-TIWI-01). Following DNA removal, the mRNA was reverse transcribed into cDNA using a reverse transcription kit (Vazyme, Nanjing, China, Catalog No. R223-01). Subsequently, RT-qPCR was performed using SYBR qPCR Master Mix (Vazyme, Nanjing, China, Catalog No. R711-03). Data analysis was conducted using the 2^−ΔΔCT^ method. The PCR primers utilized in this experiment are listed below.

- β-actin: F: GGC-TGTATTCCCCTCCATCG, R: CCAGTTGGT-AACAATGCCATGT;
- P2Y12: F: CATTGCTGTACACCGTCCTG, R: GGCTCCCAGTTTAGCATCAC;

### 4.21. Western Blot Analysis

The brain tissues were lysed in RIPA lysis buffer supplemented with phosphatase inhibitors and protease inhibitors. The protein concentration was determined using a BCA assay kit (Meilunbio, Dalian, China, MA1611). Subsequently, the protein samples (30–50 μg) were resolved by SDS-PAGE and electrotransferred onto PVDF membranes. Membranes were incubated with 5% non-fat milk in TBST for 1 h at room temperature to block non-specific binding sites prior to overnight incubation at 4 °C with primary antibodies: anti-P2Y12 (Abcam ab137752, 1:1000, Cambridge, UK), β-tubulin (CST #2146, 1:1000, Danvers, MA, USA), and β-actin (Proteintech 60008-1-Ig, 1:1000, Wuhan, China) prepared in an antibody dilution buffer (Beyotime, P0023A, Shanghai, China). The following day, the membranes were incubated with species-appropriate secondary antibodies for 1 h at room temperature. Immunolabeling was visualized using an ECL reagent (Meilunbio, Dalian, China, MA0186). Immunoblots were semi-quantitatively analyzed using ImageJ software (version 1.52a).

### 4.22. Statistical Analysis

The data were analyzed utilizing GraphPad Prism 9.5 software and are exhibited as mean ± SD. To minimize potential errors and ensure the reliability of the results, one-way ANOVA followed by Bonferroni’s post hoc test was performed for multiple comparisons. Additionally, two-way ANOVA with Bonferroni’s post hoc test was performed to compare interactions within the two-factor groups. Statistical significance was defined as *p* < 0.05.

## 5. Conclusions

This study demonstrates that DBE exhibit antithrombotic effects through the modulation of ADP-mediated platelet responses, providing neuroprotective benefits. The P2Y12 receptor signaling pathway was identified as the principal mediator of ADP-induced platelet activation. Using ADP-driven platelet aggregation models, compound **17** (Loureirin D) was identified as the core antithrombotic component of DBE, exerting its effects via the selective antagonism of the P2Y12 receptor to suppress platelet activation cascades. These findings establish a solid pharmacological basis and mechanistic insight for the development of novel antithrombotic agents.

## Figures and Tables

**Table 1:** ^1^H NMR spectroscopic data for six active compounds.

	1 ^a^	2 ^a^	7 ^a^	12 ^b^	17 ^a^	24 ^b^
pos.	*δ*_H_ (*J* in Hz)	*δ*_H_ (*J* in Hz)	*δ*_H_ (*J* in Hz)	*δ*_H_ (*J* in Hz,)	*δ*_H_ (*J* in Hz)	*δ*_H_ (*J* in Hz)
H-2a	4.19 (dd, 3.6, 11.1)	4.33 (d, 4.1, 11.4)	3.97 (d, 11.4)	3.72 (m)	3.04 (t, 2H 7.2, H-α)	6.66 (d, 2.2)
H-2b	4.03 (dd, 6.5, 11.1)	4.16 (d, 7.5, 11.4)	4.01 (d, 11.4)	4.11 (m)	2.87 (t, 2H 7.2, H-β)	-
H-3	2.64 (m)	2.77 (m)	-	2.25 (m)	5.96 (d, 2.1)	-
H-4a	-	-	-	2.21 (m)	-	6.38 (t, 2.2)
H-4b	-	-	-	2.70 (m)	-	-
H-5	-	7.75 (d, 8.7)	-	-	5.98 (d, 2.1)	-
H-6	5.95 (d, 2.1)	6.52 (dd, 1.9, 8.9)	5.91 (brs)	6.03 (d, 2.3)	-	6.66 (d, 2.2)
H-7	-	-	-	-	-	6.89 (d, 16.0)
H-8	6.06 (d, 2.0)	6.33 (d, 1.8)	5.92 (brs)	6.05 (d, 2.3)	-	7.02 (d, 16.0)
H-9a	3.01 (dd, 3.1, 12.5)	3.09 (dd, 4.3, 13.6)	2.88 (d, 14.2)	2.59 (d, 7.1)	-	-
H-9b	2.60 (m)	2.65 (d, 10.4, 13.6)	2.91 (d, 14.2)	2.59 (d, 7.1)	-	-
H-2′	7.03 (d, 8.4)	7.08 (d, 8.2)	7.08 (d, 8.3)	7.06 (d, 8.4)	7.89 (d, 8.7)	7.40 (d, 8.5)
H-3′	6.72 (d, 8.4)	6.76 (d, 8.4)	6.73 (d, 8.3)	6.78 (d, 8.4)	6.82 (d, 8.7)	6.83 (d, 8.5)
H-5′	6.72 (d, 8.4)	6.76 (d, 8.4)		6.78 (d, 8.4)	6.82 (d, 8.7)	6.83 (d, 8.5)
H-6′	7.03 (d, 8.4)	7.08 (d, 8.2)		7.06 (d, 8.4)	7.89 (d, 8.7)	7.40 (d, 8.5)
2-OMe	-	-	-	-	3.70 (s)	-
3-OMe	-	-	-	-	-	3.83 (s)
5-OMe	-	-	-	3.75 (s)	-	3.83 (s)
6-OMe	-	-	-	-	-	-
7-OMe	3.80 (s)	-	-	3.76 (s)	-	-

^a^ Recorded in MeOD. ^b^ Recorded in CDCl_3_.

**Table 2:** ^13^C NMR spectroscopic data for six active compounds.

pos.	1 ^a^	2 ^a^	7 ^a^	12 ^b^	17 ^a^	24 ^b^
1	-	-	-	-	108.1	130.2
2	69.8	70.7	72.8	69.9	160.4	104.6
3	49.9	49.8	73.5	34.0	91.9	161.1
4	194.0	195.1	199.9	25.3	157.9	99.8
4a	105.3	114.5	101.3	103.1	-	-
5	166.7	130.3	165.7	158.9	96.5	161.1
6	96.7	111.9	96.2	93.3	157.4	104.6
7	164.5	166.6	168.9	159.4	202.8	128.9
8	94.2	103.5	97.5	91.4	39.5	126.7
8a	166.4	157.1	164.4	155.9	-	-
9	33.4	33.0	40.7	37.6	19.9	-
1′	130.6	130.4	126.9	131.9	129.8	139.8
2′	131.1	131.1	132.8	130.3	132.0	128.1
3′	116.3	116.4	115.9	115.4	116.1	115.8
4′	157.0	165.3	157.5	154.1	163.6	155.6
5′	116.3	116.4	-	115.4	116.1	115.8
6′	131.1	131.1	-	130.3	132.0	128.1
2-OMe	-	-	-	-	55.8	-
3-OMe	-	-	-	-	-	55.4
5-OMe	-	-	-	55.46	-	55.4
7-OMe	56.2	-	-	55.50	-	-

^a^ Recorded in MeOD. ^b^ Recorded in CDCl_3_.

**Table 3:** Absorbed prototype components in mouse serum. (Red frame present the active component).

NO.	t (min)	Adduction	Score	Fragmentation Score	Error (ppm)	Fragments	Formula	Identification
1	7.29	M+H	72.4	89.8	−0.84	239.07034	C_15_H_10_O_3_	7-Hydroxyflavone
2	9.06	M−H	68.41	77.38	0.70	108.02054, 134.03627, 285.11298	C_17_H_18_O_4_	Loureirin A
3	11.32	M+H−H_2_O	69.93	83.68	1.22	71.04923, 113.05987, 159.11687, 251.17909, 269.19028, 281.22601, 299.23737, 375.26877, 393.27853, 411.28906	C_27_H_40_O_4_	delta5-Convallamarogenin
4	7.01	M+H	61.04	39.52	0.00	81.07001, 107.04925, 109.06492, 122.03631, 125.05981, 155.07036, 167.07037	C_9_H_10_O_3_	ISOPEONOL
5	5.39	M−H	49.01	71.28	1.06	240.04222, 268.03751, 283.06082	C_16_H_12_O_5_	Kakkatin
6	7.33	M+H	54.85	72.43	0.00	107.04926, 181.04967, 287.09149	C_16_H_14_O_5_	(2R,3R)-3,7-dihydroxy-6-methoxyflavanone
7	6.05	M+H	50.1	95.38	0.00	141.05478, 149.05992, 153.05475	C_16_H_16_O_5_	Loureirin D
8	11.13	M+Na	44.24	88.06	−8.47	135.1168, 147.1167, 149.1327, 151.1120, 161.1327, 179.1434, 231.2109, 241.1952, 259.2061, 277.2164	C_16_H_32_O_3_	Ethyl,alpha-hydroxymyristate
9	7.79	M+H	58.93	94.82	0.33	149.05992, 155.07033, 167.07043	C_17_H_18_O_5_	phenethyl,vanillate
10	6.07	M−H	46.22	93.79	7.60	93.03344, 139.03909, 147.0442, 151.03912, 229.13539, 287.09241, 309.07419	C_19_H_16_O_7_	2′-hydroxymethylophiopogonone a
11	11.13	M+H	53.28	42.98	7.25	317.20886	C_20_H_28_O_3_	(+)-Hardwickiic acid
12	11.02	M−H	48.19	54.92	7.67	183.13811, 297.24316, 319.22525, 365.23047	C_21_H_34_O_5_	Isomuronic acid
13	4.79	M+H	66.23	41.10428571	−0.63	121.0286, 147.0807, 181.08615, 287.09134, 319.11761	C_17_H_18_O_6_	10-o-methyl protosappanin b
14	8.00	M+FA−H	48.95	66.55	0.91	135.04402, 139.03899, 147.0441, 151.03899, 287.09222, 299.09216, 439.13956	C_23_H_22_O_6_	(+)-purpurin 2
15	13.69	M+FA−H	67	89.95	0.45	403.35791, 447.34766	C_27_H_46_O_2_	Furostanol
16	8.95	M+H	48.46	64.76	0.00	245.15349, 337.25281, 355.26303, 373.27371	C_24_H_36_O_3_	2-[(9Z,12Z)-heptadeca-9,12-dienyl]-6-hydroxybenzoic acid
17	9.04	M−H	49.59	70.75	0.37	311.1279, 312.1311, 313.1052, 313.1357, 377.1388, 395.1472, 403.1552, 449.1569, 497.1968, 543.2028	C_32_H_32_O_8_	nobiline
18	9.91	M+NH4	74.06	93.56	−0.46	107.08563, 119.0857, 135.08054, 229.87602, 281.13885, 415.21146	C_24_H_30_O_6_	caesaldekarin e
19	9.14	M+Cl	45.14	82.07	−3.24	93.03341, 135.04408, 321.07629, 415.11832, 555.16656	C_26_H_32_O_11_	camaldulenside
20	7.80	M+H	52.81	90.86	0.21	137.05981	C_30_H_28_O_6_	marchantin k
21	9.21	M+K	49.32	85.34	−3.68	137.05984	C_28_H_40_O_8_	TAXUSIN
22	10.16	M+K	42.45	56.53	5.70	73.02848, 251.17941, 269.19003, 287.20056, 419.24283, 561.34241	C_33_H_50_N_2_O_3_	pachysandrine a
23	9.17	M+Na	53.56	94.1	0.61	339.12045	C_36_H_40_O_10_	orthosiphonone d

## Data Availability

The original contributions presented in this study are included in the article. Further inquiries can be directed to the corresponding author.

## Abbreviations

AA, arachidonic acid; ACD, acid citrate-dextrose; ADP, adenosine diphosphate; BBB, blood–brain barrier; BCA, bicinchoninic acid; CC, column chromatography; CETSA, cellular thermal shift assay; CH_2_Cl_2_, dichloromethane; CLP, clopidogrel; COX, cyclooxygenase; DBE, Dragon’s Blood phenolic extracts; EA, ethyl acetate; ECL, enhanced chemiluminescence; FeCl_3_, ferric chloride; HPLC, high-performance liquid chromatography; HT, hemorrhagic transformation; MeOH, methanol; mNSS, modified Neurological Severity Score; P2Y12, purinergic receptor P2Y12; PE, petroleum ether; PRP, platelet-rich plasma; PT-stroke, photothrombotic stroke; PVDF, polyvinylidene fluoride; RAPA, rapamycin; SDS-PAGE, sodium dodecyl sulfate-polyacrylamide gel electrophoresis; TXA_2_, thromboxane A_2_.
